# An individualized medication model of sodium valproate for patients with bipolar disorder based on machine learning and deep learning techniques

**DOI:** 10.3389/fphar.2022.890221

**Published:** 2022-10-17

**Authors:** Ping Zheng, Ze Yu, Liqian Mo, Yuqing Zhang, Chunming Lyu, Yongsheng Yu, Jinyuan Zhang, Xin Hao, Hai Wei, Fei Gao, Yilei Li

**Affiliations:** ^1^ Department of Pharmacy, Nanfang Hospital, Southern Medical University, Guangzhou, China; ^2^ Institute of Interdisciplinary Integrative Medicine Research, Shanghai University of Traditional Chinese Medicine, Shanghai, China; ^3^ Zhongshan School of Medicine, SYSU, Guangzhou, China; ^4^ Experiment Center for Science and Technology, Shanghai University of Traditional Chinese Medicine, Shanghai, China; ^5^ Beijing Medicinovo Technology Co., Ltd., Beijing, China; ^6^ Dalian Medicinovo Technology Co., Ltd., Dalian, Liaoning, China

**Keywords:** sodium valproate, individualized dose, machine learning, deep learning, CatBoost, bipolar disorder

## Abstract

Valproic acid/sodium valproate (VPA) is a widely used anticonvulsant drug for maintenance treatment of bipolar disorders. In order to balance the efficacy and adverse events of VPA treatment, an individualized dose regimen is necessary. This study aimed to establish an individualized medication model of VPA for patients with bipolar disorder based on machine learning and deep learning techniques. The sequential forward selection (SFS) algorithm was applied for selecting a feature subset, and random forest was used for interpolating missing values. Then, we compared nine models using XGBoost, LightGBM, CatBoost, random forest, GBDT, SVM, logistic regression, ANN, and TabNet, and CatBoost was chosen to establish the individualized medication model with the best performance (accuracy = 0.85, AUC = 0.91, sensitivity = 0.85, and specificity = 0.83). Three important variables that correlated with VPA daily dose included VPA TDM value, antipsychotics, and indirect bilirubin. SHapley Additive exPlanations was applied to visually interpret their impacts on VPA daily dose. Last, the confusion matrix presented that predicting a daily dose of 0.5 g VPA had a precision of 55.56% and recall rate of 83.33%, and predicting a daily dose of 1 g VPA had a precision of 95.83% and a recall rate of 85.19%. In conclusion, the individualized medication model of VPA for patients with bipolar disorder based on CatBoost had a good prediction ability, which provides guidance for clinicians to propose the optimal medication regimen.

## Introduction

Valproic acid/sodium valproate (VPA) constitutes a widely prescribed anticonvulsant drug for maintenance treatment of bipolar disorders. VPA is a short-chain fatty acid that acts as a gamma-aminobutyric acid transaminase inhibitor and blocker of voltage-gated sodium channels and T-type calcium channels and inhibiting histone deacetylase, which impacts systems associated with manic-type behaviors (Perucca, 2002; Bowden and Karren, 2006; Chateauvieux et al., 2010). Bipolar disorder is a recurrent illness characterized by depressive and manic/hypomanic episodes, affecting more than 1% of the population worldwide (Cipriani et al., 2013; Pisanu et al., 2018; Fontana et al., 2020). VPA is effective for treating mania, alone or in combination (Bowden and Karren, 2006). Wide variability in VPA pharmacokinetic parameters exists in patients with bipolar disorder, and they normally need long anticipated treatment durations (Haymond and Ensom, 2010). Some common side effects of VPA include alopecia, tremor, and weight gain (Cipriani et al., 2013). When the acceptability of long-term treatment is considered, together with efficacy and adverse events induced by VPA, an individualized dose regimen is important.

Compared with conventional modeling methods, machine learning and deep learning techniques have indubitable advantages in dealing with real-world data, such as 1) machine learning and deep learning techniques can deal with more complex, high-dimensional, and interactive variables, which is lacking in traditional models; 2) machine learning and deep learning models have stronger generalization and better accuracy than conventional models (Kruppa et al., 2012; Lee et al., 2018; Mo et al., 2019). Recently, some algorithms with more sophisticated principles have been developed, such as eXtreme Gradient Boosting (XGBoost), light gradient boosting machine (LightGBM), categorical boosting (CatBoost), and gradient boosting decision tree (GBDT), which have been highly recognized in algorithm competitions (Chen and Guestrin, 2016; Ke et al., 2017; Prokhorenkova et al., 2017; Zhang et al., 2019). CatBoost is designed for processing classification features based on the modification of a standard gradient boosting algorithm, which uses binary decision trees as base predictors (Prokhorenkova et al., 2017). In medical and pharmacy application, CatBoost is widely used in various classification or regression studies with promising prediction results, such as the prediction of fractures (Kong et al., 2020). Recently, the application of machine learning and deep learning techniques on individualized medication models has been approbatory, such as a novel vancomycin dose prediction model through XGBoost and warfarin maintenance dose prediction through LightGBM (Huang et al., 2021; Liu et al., 2021). With the increasing number of input subject data, the model can continually optimize parameters to achieve better accuracy and practicality.

In order to achieve a balance of drug efficacy and toxicities, an appropriate dose regimen is important for the patient’s treatment outcome. In this study, we aimed to establish a machine learning or deep learning model to predict the VPA daily dose based on important influencing variables, resulting in obtaining the optimal individualized dose regimen with high predictive abilities.

## Materials and methods

### Study population

We enrolled patients who were diagnosed with bipolar disorder and treated with VPA at the Southern Medical University Nanfang Hospital from 17 July 2018, to 12 December 2021. Pregnant or lactating female patients were excluded. Study data have been fully deidentified, and confidential information of patients has been deleted, in accordance with the CIOMS/WHO International Ethical Guidelines for Health-related Research Involving Humans (2016). Consequently, the study was deemed exempt from informed consent by study participants.

### Data collection and cleaning

**FIGURE 1:**
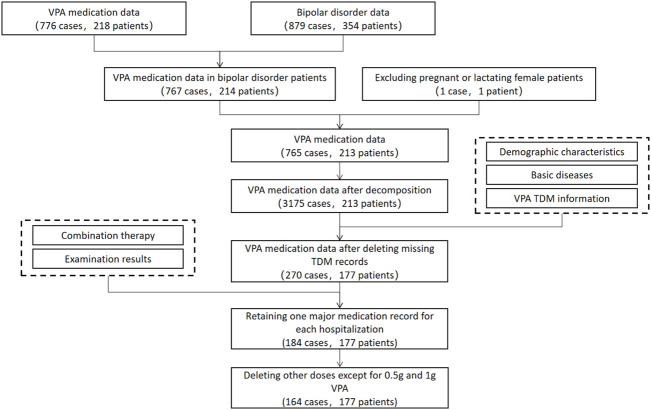
Enrollment of patients.

All data were collected from patients’ electronic medical records and were cleaned (Figure 1). First, we extracted VPA data from doctor orders after combining multiple VPA orders on a daily basis and deleting repeated orders and 218 patients with 776 cases of data were obtained. On the other hand, we extracted diagnosis data of bipolar disorder from diagnostic records and obtained 354 patients with 879 cases of data. The data of patients with bipolar disorder were extracted from VPA data, and we obtained 214 patients with 767 cases of data. After deleting data of pregnant or lactating female patients, there were 213 patients with 765 cases of data. Subsequently, the daily dose of VPA was calculated, and units of dosage and frequency were unified (daily dose = dosage * frequency). Temporary and long-term VPA orders were combined, and long-term VPA orders were broken down into one record per day. To be specific, if temporary VPA orders existed on the day of long-term VPA orders, only the daily dose in the temporary VPA order was adopted; otherwise, the daily dose in the long-term VPA order was adopted; if VPA was prescribed in the morning, a daily dose of the whole day was adopted; if VPA was prescribed in the afternoon, 1/2 or 1/3 of the daily dose was adopted; and, if the time of the temporary VPA order was during the period of the long-term VPA order, the daily dose of the temporary order was added to the long-term order. After data decomposition, 213 patients with 3,175 cases of data were obtained. Among these, demographic characteristics (including gender, age, height, and weight) and basic disease information (including hypertension, diabetes, cerebrovascular disease, hepatitis, and kidney disease) were extracted from patient’s records, and VPA TDM information was extracted from laboratory data (if VPA was administrated within four days prior to TDM, the latest TDM record was adopted). After deleting missing TDM records, 177 patients with 270 cases of data were obtained. Other test results within 4 four days prior to VPA TDM were extracted from laboratory data. Combination therapy information within four days prior to VPA TDM was extracted from doctor orders, including anticonvulsants and mood stabilizers (such as carbamazepine, oxcarbazepine, and lamotrigine), antipsychotics (such as olanzapine, quetiapine, aripiprazole, ziprasidone, risperidone, paliperidone, clozapine, and chlorpromazine), antidepressants (such as photioxetine hydrobromide, agomelatine, citalopram, sertraline, escitalopram, fluoxetine, paroxetine, mirtazapine, and trazodone), antianxiety drugs/tranquilizers/hypnotic drugs (such as clonazepam, diazepam, lorazepam, alprazolam, dospirone, dexzopiclone, zolpidem, shumian capsule, wuling capsule, and estazolam), and behavioral therapy (suggestive therapy, guided education and training, behavior correction therapy, and impulsive behavior intervention therapy) (Hiemke et al., 2011; Hiemke et al., 2018). After deleting examination results with a large amount of missing data and retaining one major medication record for each hospitalization, 177 patients with 184 cases of data were obtained. Herein, the individualized medication model of VPA mainly predicted daily doses of 0.5 and 1 g, after deleting 20 cases of other doses, finally corresponding to 177 patients with 164 cases of data.

### Data processing

**FIGURE 2:**
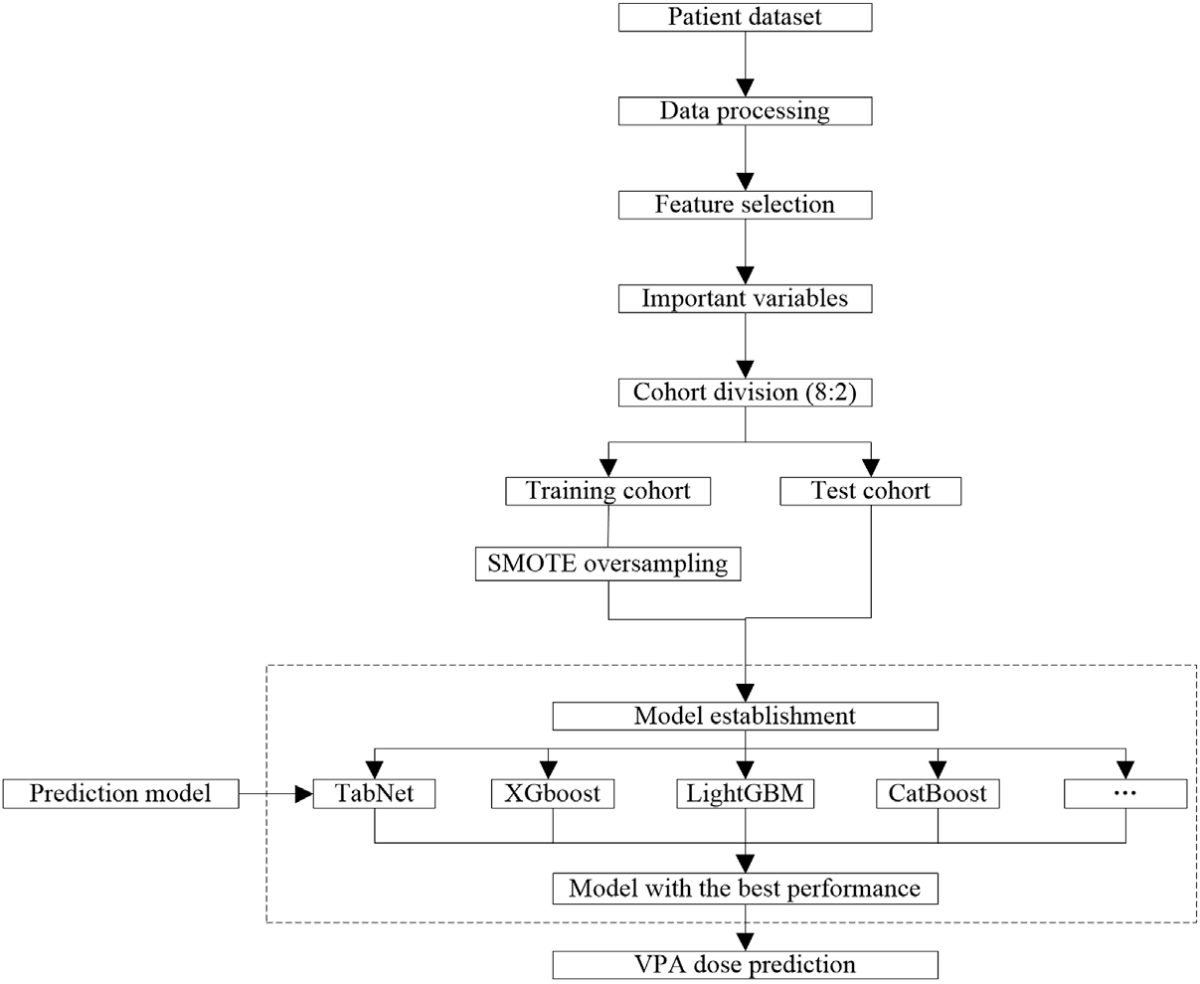
Workflow of data processing and modeling.

In order to improve the accuracy of the individualized medication model, medication data of VPA were preprocessed (Figure 2). Specifically, a binary variable was dealt with one-hot encoding. Irrelevant variables (such as patient ID, case no., drug administration time, and hospitalization time), missing rates over 50% and extremely unbalanced variables, and abnormal values were deleted. After that, the sequential forward selection (SFS) algorithm was applied for feature engineering to select the minimum size and optimum performance of the feature subset (Hatamikia et al., 2014). The SFS algorithm added one feature to the feature subset each time, iteratively generated a new model, and calculated the model performance [area under the curve (AUC)]. In order to ensure data integrity, the linear model and nonlinear model are trained, the VPA dose of patients with bipolar disorder is predicted, and the variables screened by feature engineering were interpolated with missing values based on random forest. Subsequently, the training cohort and test cohort were divided according to 8:2. Due to the unbalanced distribution of the VPA dose, it is necessary to adopt over-sampling on the training cohort to learn useful information from unbalanced data sets and improve the prediction ability of the model. As the daily dose of 0.5 g VPA has a low proportion in the dataset, we applied the SMOTE oversampling algorithm to generate a different number of low-dose samples (Chawla et al., 2002).

### Model establishment

In this study, the daily dose of VPA was set as the target variable, and a daily dose of 0.5 g corresponds to “0,” and a daily dose of 1 g corresponds to “1.” Based on the selected features, we established a linear model and nonlinear model and obtained the prediction performance of different models after parameter adjustment (Figure 2). Herein, we compared the model performance of nine algorithms, namely, XGBoost, LightGBM, CatBoost, random forest, GBDT, support vector machine (SVM), logistic regression, artificial neural network (ANN), and TabNet. The dose prediction performance of all models was evaluated through precision, recall, F1-score, accuracy, sensitivity, specificity, and AUC. Ultimately, the model with the best evaluating indexes was selected as the final model to predict the VPA dose.

### Clinical interpretation

The importance of variables refers to the degree to which each variable in the model contributes to improving the predictive power of the whole model. We calculated and ranked the importance scores of variables using the algorithm with the best predictive performance. Variables with higher importance scores were more closely related to the accurate prediction of VPA dose. Afterward, we used the SHapley Additive exPlanations (SHAP) to visually interpret the impacts of important variables on the model output (Lundberg and Lee, 2017). SHAP could help explain which variables have positive or negative impacts on predicting the VPA dose. Eventually, a confusion matrix was used to visualize the performance of the algorithm and further analyze the model performance in the test cohort.

## Results

### Baseline information

The study population’s baseline information is shown in Table 1. The continuous variables were described by “mean (interquartile range, IQR)”, while the classification variables were described by “frequency (percentage, %).” There were 29 (17.68%) patients administered with a daily VPA dose of 0.5 g, and 135 (82.32%) patients administered with a daily VPA dose of 1 g. The mean VPA TDM value was 79.84 (IQR 62.77–95.45) ng/ml. The mean age of cases in this study was 25.71 (IQR 18.00–29.00) years, the proportion of female patients was 53.66%, the mean height was 165.39 (IQR 159.00–170.25) cm, the mean weight was 61.16 (IQR 52.00–71.25) kg, and the mean body mass index was 22.25 (IQR 19.34–24.67) kg/m^2^. Patients with basic diseases occupied 7.93%. The percentage of those using combination therapies was 1.83% for anticonvulsants and mood stabilizers, 72.56% for antipsychotics, 13.61% for antidepressants, 13.61% for antianxiety drugs/tranquilizers/hypnotic drugs, and 80.49% for behavioral therapy.

**TABLE 1:** Description of demographic and clinical characteristics.

Categories	Variables		Cases (N = 164)	Missing rate (%)
Target variable	VPA daily dose, g, n (%)	0.5	29 (17.68%)	0
		1	135 (82.32%)	0
VPA information	VPA TDM, ng/ml, median (IQR)		79.55 (62.77–95.45)	0
Demographic information	Age, y, median (IQR)		23.00 (18.00–29.00)	0
	Gender, n (%)	Male	76 (46.34%)	0
		Female	88 (53.66%)	0
	Height, cm, median (IQR)		164.50 (159.00–170.25)	0
	Weight, kg, median (IQR)		58.00 (52.00–71.25)	12.80
	BMI, kg/m^2^, median (IQR)		21.91 (19.34–24.67)	12.80
Basic disease	Basic disease, n (%)		13 (7.93%)	0
Combination	Anticonvulsants and mood stabilizers, n (%)		3 (1.83%)	0
	Antipsychotics, n (%)		119 (72.56%)	0
	Antidepressants, n (%)		22 (13.61%)	0
	Antianxiety drugs/tranquilizers/hypnotic drugs, n (%)		22 (13.61%)	0
	Behavioral therapy, n (%)		132 (80.49%)	0
Essay index	PLT, ×10^9/L, median (IQR)		225.00 (200.50–260.00)	44.51
	P-LCR, %, median (IQR)		26.20 (22.55–33.45)	44.51
	MCH, pg, median (IQR)		29.60 (28.65–30.60)	44.51
	BASO, %, median (IQR)		0.60 (0.40–0.90)	44.51
	BUN, mmol/L, median (IQR)		3.70 (3.10–4.55)	49.39
	LYM, %, median (IQR)		36.70 (31.75–42.25)	44.51
	RDW-CV, %, median (IQR)		12.50 (12.00–13.25)	44.51
	AST, U/L, median (IQR)		16.00 (13.00–21.50)	46.95
	PCT, %, median (IQR)		2.00 (1.77–2.28)	44.51
	PDW, %, median (IQR)		16.10 (15.70–16.40)	44.51
	MONO, ×10^9/L, median (IQR)		0.45 (0.38–0.53)	44.51
	WBC, ×10^9/L, median (IQR)		6.26 (5.46–7.38)	44.51
	NEU, %, median (IQR)		52.10 (45.70–58.40)	44.51
	MPV, fL, median (IQR)		8.90 (8.20–10.15)	44.51
	MCV, fL, median (IQR)		90.00 (86.95–92.50)	44.51
	EOS, ×10^9/L, median (IQR)		0.14 (0.09–0.26)	44.51
	RBC, ×10^9/L, median (IQR)		4.60 (4.22–5.02)	44.51
	A/G, median (IQR)		1.50 (1.40–1.60)	46.95
	NEU, ×10^9/L, median (IQR)		3.15 (2.62–3.94)	48.78
	Hb, g/L, median (IQR)		132.00 (123.00–145.00)	44.51
	RDW-SD, %, median (IQR)		42.20 (40.10–44.35)	44.51
	LYM, ×10^9/L, median (IQR)		2.28 (1.79–2.96)	44.51
	MONO, %, median (IQR)		7.00 (6.20–8.45)	44.51
	UA, μmol/L, median (IQR)		372.00 (313.50–462.00)	48.78
	Cr, μmol/L, median (IQR)		66.00 (55.00–75.00)	49.39
	Albumin, g/L, median (IQR)		40.60 (38.35–42.85)	46.95
	Transaminase ratio, median (IQR)		1.00 (0.80–1.50)	46.95
	MCHC, g/L, median (IQR)		329.00 (323.50–332.00)	44.51
	HCT, L/L, median (IQR)		0.41 (0.38–0.44)	44.51
	EOS, %, median (IQR)		2.20 (1.70–4.10)	44.51
	Indirect bilirubin, μmol/L, median (IQR)		4.90 (3.70–6.50)	46.95
	Total bilirubin, μmol/L, median (IQR)		7.60 (5.75–9.65)	46.95
	Globulin, g/L, median (IQR)		26.90 (25.00–29.65)	46.95
	Direct bilirubin, μmol/L, median (IQR)		2.70 (2.00–3.80)	46.95
	TP, g/L, median (IQR)		68.00 (63.95–72.30)	46.95
	BASO, ×10^9/L, median (IQR)		0.04 (0.03–0.05)	44.51

Abbreviations: VPA, valproic acid; IQR, interquartile range; TDM, therapeutic drug monitoring; BMI, body mass index; PLT, platelet; P-LCR, platelet-large cell rate; MCH, mean corpuscular hemoglobin; BASO, basophil; BUN, blood urea nitrogen; LYM, lymphocyte; RDW-CV, red cell distribution width-coefficient of variation; AST, aspartate aminotransferase; PCT, platelet hematocrit; PDW, platelet distribution width; MONO, monocyte; WBCs, white blood cells; NEU, neutrophil; MPV, mean platelet volume; MCV, mean corpuscular volume; EOS, eosinophils; RBCs, red blood cells; A/G, albumin/globulin; Hb, hemoglobin; RDW-SD, red cell distribution width-standard deviation; UA, uric acid; MCHC, mean corpuscular hemoglobin concentration; HCT, hematocrit; TP, total protein.

### Variable analysis

**FIGURE 3:**
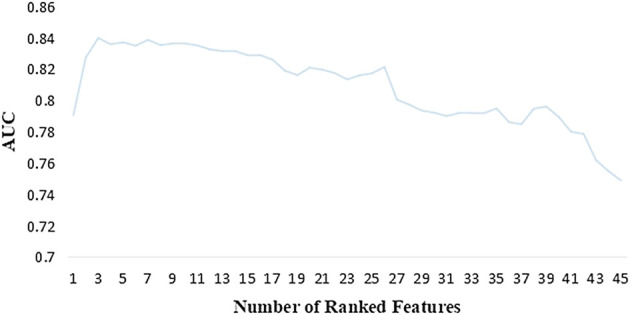
AUC of the XGBoost model corresponding to the number of ranked features.

After data preprocessing, features were selected based on 45 variables through the SFS method. XGBoost models were established using the selected one to 45 variables, and the AUC of each model was obtained (Figure 3). With an increasing number of included variables, the AUC value keeps increasing, reaches its maximum value when three variables were selected (AUC = 0.839), and then decreases. As we pursued a concise and accurate model with minimal variables but the highest predictive performance, the first three important variables were selected to establish the individualized medication model, i.e., VPA TDM value, antipsychotics, and indirect bilirubin.

### Model performance and interpretation

Models were established by nine algorithms, and modeling parameters are illustrated in Supplementary Table S1. The parameters of the CatBoost model were iterations = 300, learning_rate = 0.02, depth = 6, l2_leaf_reg = 2, subsample = 1, loss_function = 'cross-entropy’, and random_state = 3. In Table 2, we present the prediction performance of nine models. CatBoost had precision = 0.56, recall = 0.83, and F1_score = 0.67 for predicting the daily dose of 0.5 g VPA; precision = 0.96, recall = 0.85, and F1_score = 0.90 for predicting the daily dose of 1 g VPA; and accuracy = 0.85, AUC = 0.91, sensitivity = 0.85, and specificity = 0.83 for the whole CatBoost model, the metrics of which were higher than those of other algorithms and achieved a best comprehensive performance. Therefore, CatBoost was selected to predict the daily dose of VPA and subsequently to calculate the importance scores of variables and analyze the dose prediction effect.

**TABLE 2:** Prediction performance of different algorithms.

Metrics algorithms	Dose regimen^a^	Precision	Recall	f1_score	Accuracy	AUC	Sensitivity	Specificity
XGBoost	0	0.45	0.83	0.59	0.79	0.87	0.78	0.83
	1	0.95	0.78	0.86				
LightGBM	0	0.50	0.67	0.57	0.82	0.84	0.85	0.67
	1	0.92	0.85	0.88				
CatBoost	0	0.56	0.83	0.67	0.85	0.91	0.85	0.83
	1	0.96	0.85	0.90				
Random forest	0	0.40	0.67	0.50	0.76	0.88	0.78	0.67
	1	0.91	0.78	0.84				
GBDT	0	0.29	0.33	0.31	0.73	0.77	0.81	0.33
	1	0.85	0.81	0.83				
SVM	0	0.44	0.67	0.53	0.79	0.82	0.81	0.67
	1	0.92	0.81	0.86				
Logistic regression	0	0.44	0.67	0.53	0.79	0.81	0.81	0.67
	1	0.92	0.81	0.86				
ANN	0	0.40	0.67	0.50	0.76	0.85	0.78	0.67
	1	0.91	0.78	0.84				
TabNet	0	0.44	0.67	0.53	0.79	0.8	0.81	0.67
	1	0.92	0.81	0.86				

^a^ Regimen of the daily dose of 0.5 g VPA corresponds to “0,” and regimen of the daily dose of 1 g VPA corresponds to “1”.

On this basis, the importance scores of three selected variables were calculated and ranked by CatBoost (Table 3). Among them, the importance score of the VPA TDM value was remarkably higher than that of the other two variables (importance score = 56.048), followed by antipsychotics (importance score = 26.479) and indirect bilirubin (importance score = 17.473). A higher importance score indicates the greater impact of this variable on the prediction of VPA daily dose.

**TABLE 3:** Importance scores of variables.

No.	Variables	Importance score
1	VPA TDM value	56.048
2	Antipsychotics	26.479
3	Indirect bilirubin	17.473

**FIGURE 4:**
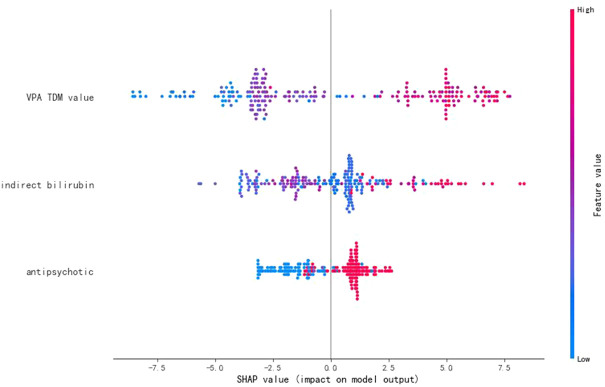
SHAP values of the important variables. The dot color is redder when the feature value gets higher and bluer when the feature value gets lower. When the SHAP value gets higher, the impact of the variable on model output is larger.

SHAP values represent the impacts on model output, which is the prediction of VPA daily dose (Figure 4). The feature value means the contribution of each variable to the predictive power of the model. For the VPA TDM value, antipsychotics, and indirect bilirubin, the dot color is redder when the SHAP value becomes larger, while it is bluer when the SHAP value becomes smaller, thus showing the positive impacts of these variables on VPA daily dose.

**FIGURE 5:**
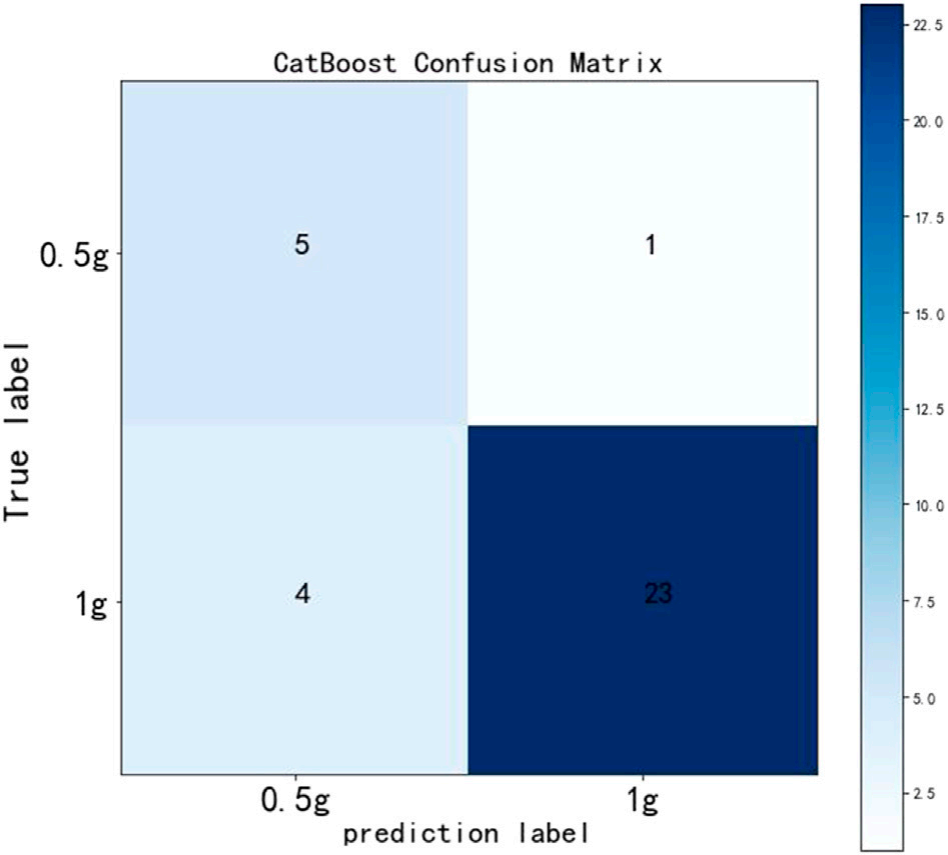
Confusion matrix in the CatBoost model.

The test cohort consisted of 33 patients, among which, six patients took a daily dose of 0.5 g VPA and 27 patients took a daily dose of 1 g VPA. The dose of VPA was recommended for patients by establishing a confusion matrix based on the CatBoost prediction model (Figure 5). The model recommended a daily dose of 1 g VPA for 24 patients, including one patient who was recommended the wrong dose, with a precision of 95.83% and a recall rate of 85.19%; the model recommended a daily dose of 0.5 g VPA for nine patients, including four patients who were recommended the wrong dose, with a precision of 55.56% and recall rate of 83.33%.

## Discussion

Our study focused on the establishment of an individualized medication model for VPA daily dose in patients with bipolar disorder, mainly evaluating two daily dose regimens (0.5 and 1 g). We used CatBoost, a leading-edge machine learning method based on the upgraded gradient boosting algorithm, to construct the prediction model with good performance (accuracy = 0.85, AUC = 0.91, sensitivity = 0.85, and specificity = 0.83). Afterward, important variables that strongly correlated with VPA daily dose were ranked *via* the importance score, including VPA TDM value, antipsychotics, and indirect bilirubin. Last, a confusion matrix was used to validate the model, and it can be observed that predicting a daily dose of 0.5 g VPA had a precision of 55.56% and a recall rate of 83.33%, and predicting a daily dose of 1 g VPA had a precision of 95.83% and a recall rate of 85.19%.

VPA has a wide range of metabolic pathways. Approximately 40% of the dose is metabolized in the pathway of mitochondrial beta-oxidation, producing 2-enevalproate and 3-keto-valproate (Silva et al., 2008; Tomson et al., 2013). About 10%–15% of the dose is eliminated through a metabolic pathway catalyzed by CYP enzymes, forming hydroxylated metabolites (3-, 4-, and 5-hydroxyvalproate metabolites) and 4-enevalproate (Sadeque et al., 1997; Levy et al., 2002; Kiang et al., 2006). When adults receive monotherapy of VPA, its conjugation with glucuronic acid is the major metabolic pathway, and 30%–50% of the dose is converted to valproate glucuronide, about 20% of which is excreted in urine (Besag and Berry, 2006; Monostory et al., 2019).

VPA is associated with non-linear pharmacokinetics because of saturable plasma protein binding, resulting in large individual differences in the dose-to-plasma concentration relationship (Patsalos et al., 2018). Dosing of VPA benefits from serum level determinations (Bowden and Karren, 2006). Previous studies on manic episodes found that serum levels above 45 μg ml^−1^ led to higher drug response rates (Bowden and Karren, 2006). Currently, clinicians usually make dose adjustments based on TDM results, showing a positive interaction between VPA daily dose and the TDM value in our study, as a critical predictor in the individualized medication model of VPA.

VPA is susceptible to many drug–drug pharmacokinetic interactions because its metabolism is readily induced or inhibited by multiple combined used drugs (Patsalos et al., 2018). VPA is typically used in combination with antipsychotics to reduce the symptoms of epilepsy, bipolar disorder, schizophrenia, and schizoaffective disorder (Casey et al., 2003). The augmentation of antipsychotics with VPA resulted in better clinical response, especially the improvement of excitement and aggression (Wang et al., 2016). In terms of specific antipsychotics, some studies found inconsistent information about the interactions between VPA and these antipsychotics. For instance, van Wattum suggested in a case report that risperidone might increase VPA concentration, whereas Vitiello found that risperidone possibly decreased VPA serum concentrations by 30% (van Wattum, 2001; Vitiello, 2001). In addition, chlorpromazine appeared to inhibit the metabolism of VPA and possibly increased its concentration, but the reliability of supporting clinical data was low (Mula and Monaco, 2002). Moreover, VPA was deemed as both an inducer and a competitive inhibitor of olanzapine metabolism, and the interactions between clozapine and VPA, aripiprazole, and VPA were still disputable (Spina et al., 2016). In our study, the usage of antipsychotics was positively correlated with that of VPA daily dose, contributing to an important predictor in the VPA dose prediction model. In future, the mechanisms and clinical relevance of these drug interactions need to be better-investigated.

Furthermore, VPA is extensively metabolized in the liver, and its reactive metabolites have been reported to be associated with hepatotoxicity (Monostory et al., 2019; Zhang et al., 2020). Indirect bilirubin, the abnormal value of which indicates abnormal liver function, had a positive relationship with VPA daily dose as shown in our results. In the individualized medication model of VPA, indirect bilirubin, as a remarkable predictor, can help clinicians adjust the VPA dose regimen reasonably.

CatBoost is a new GBDT algorithm that can deal with categorical features well. Characteristics of the CatBoost algorithm include the following: 1) CatBoost allows the use of the whole dataset for training. Target statistics is an efficient method for handling categorical features with minimum information loss. Specifically, for each example, the input sample set is randomly sorted, and multiple groups of random permutations are generated. Floating point or attribute value tokens are converted to integers. All the classification eigenvalue results are transformed into numerical results according to the following formula: $x_{\delta_{n,k}}=\frac{\overset{n-1}{\underset{j=1}{\sum }}{[x}_{\delta_{j,k}}=x_{\delta_{n,k}}]\cdot y+\varepsilon \cdot p}{\overset{n-1}{\underset{j=1}{\sum }}{[x}_{\delta_{j,k}}=x_{\delta_{n,k}}]+\varepsilon }$ , where *p* is a prior value and ϵ is the weight of the prior. 2) Feature combinations: all classification features can be combined into a new classification feature. When a new split of the tree is performed, the features are combined in a greedy manner, and all the combinations of classification features and designs are combined to form a new feature. 3) Overcoming gradient bias: CatBoost has a unique calculation method of leaf value. The first stage of CatBoost uses an unbiased estimation of gradient step size, and the second stage is executed using the traditional GBDT scheme. 4) Fast scorer: Catboost always uses a full binary tree, and its nodes are mirrored to avoid overfitting, increase reliability, and greatly accelerate the prediction. CatBoost uses the oblivious tree as the base predictor, which is balanced and reduces overfitting. The first step is to binarize all floating point features, statistics, and one-hot encoding features. The second step is to use the binary features to calculate model predictions (Prokhorenkova et al., 2017; Hancock and Khoshgoftaar, 2020).

One advantage of our study is the application of machine learning and deep learning techniques for individualized medication modeling, which can deeply mine data from the clinical medical center. Second, we compared multiple models and selected CatBoost with the best predictive abilities, which provided the reasonability of the modeling process. Moreover, we adopted rigorous data processing techniques, such as the SFS algorithm to select minimal feature subsets with optimal performance and SMOTE oversampling algorithm, to fully utilize the unbalanced dataset. Last, we selected three predictors, namely, VPA TDM value, antipsychotics, and indirect bilirubin, to establish a robust individualized medication model of VPA based on powerful evidence, which few studies investigated before. One limitation is that we used a limited sample size from only one medical center, which necessitates a larger sample size from multiple medical centers in the future, to increasingly improve model performance by adding new data. Another limitation is the shortage of considering the impact of gene polymorphism on VPA dose, such as CYP2C9, which can be taken into account in future research.

To conclude, this study was designed to mine in-depth influencing factors to predict VPA daily dose in patients with bipolar disorder. In our study, a machine learning technique, CatBoost, was adopted to establish the dose prediction model with the best performance. The application of an individualized medication model for VPA daily dose provides guidance and recommendations for clinicians to propose the optimal medication regimen.

## Data Availability

The raw data supporting the conclusion of this article will be made available by the authors, without undue reservation.
