# Supplementary material for: An individualized medication model of sodium valproate for patients with bipolar disorder based on machine learning and deep learning techniques
Source: Front Pharmacol. 2022 Oct 17;13:890221. doi: 10.3389/fphar.2022.890221 (PMC9627622; doi:10.3389/fphar.2022.890221)
Supplement: Supplementary file 1 [file Table1.DOCX]

**Supplementary Table S1. Modeling parameters of 9 models**

| **Model** | **Parameter** |
| --- | --- |
| XGboost | max_depth=5,  learning_rate=0.1,  n_estimators=500,  min_child_weight=0.6, eta=0.1,  gamma=0.5,  reg_lambda=8,  subsample=0.5,  colsample_bytree=0.9,  nthread=4,  scale_pos_weight=1,  random_state=3 |
| LightGBM | iterations=300,  max_depth=8,  min_child_weight=0.9,  gamma=0.5,  reg_lambda=5,  subsample=0.4,  learning_rate=0.3,  loss_function='CrossEntropy',  random_state=3 |
| CatBoost | iterations=300,  learning_rate=0.02,  depth=6,  l2_leaf_reg=2,  subsample=1,  loss_function='CrossEntropy',  random_state=3 |
| RF | n_estimators=150,  max_depth=8,  random_state=3 |
| GBDT | n_estimators=300,  learning_rate=0.1,  max_depth=8,  subsample=0.4,  random_state=3 |
| SVM | kernel='rbf',  C=50,  cache_size=200,  probability=True,  random_state=3 |
| LR | penalty='l2',  C=5,  solver='lbfgs',  max_iter=100,  random_state=3 |
| ANN | alpha=0.1,  hidden_layer_sizes=[100,],  solver='adam',  activation='relu',  random_state=3 |
| TabNet | n_d=8,  n_a=8,  n_steps=3, gamma=1.5,  n_independent=2  eval_metric=['auc'],  max_epochs=200,  patience=50,  batch_size=128,  virtual_batch_size=14,  num_workers=0,  drop_last=False |
